# Long-term costs of colorectal cancer treatment in Spain

**DOI:** 10.1186/s12913-016-1297-6

**Published:** 2016-02-16

**Authors:** Julieta Corral, Xavier Castells, Eduard Molins, Pietro Chiarello, Josep Maria Borras, Francesc Cots

**Affiliations:** Department of Pediatrics, Obstetrics and Gynecology, Preventive Medicine and Public Health, Doctoral Programme in Public Health, Universitat Autònoma de Barcelona, Barcelona, Spain; Department of Health, Catalonian Cancer Strategy, Generalitat de Catalunya, Barcelona, Spain; IMIM (Hospital del Mar Medical Research Institute), Barcelona, Spain; Health Services Research on Chronic Patients Network (REDISSEC), Barcelona, Spain; Department of Statistics and Operations Research, Universitat Politècnica de Catalunya, Barcelona, Spain; Department of Clinical Sciences, Bellvitge Biomedical Research Institute (IDIBELL), Universitat de Barcelona, Barcelona, Spain

**Keywords:** Colorectal cancer, Health care, Hospital, Cost analysis, Long-term cost, Incidence-based

## Abstract

**Background:**

Assessing the long-term cost of colorectal cancer (CRC) increases our understanding of the disease burden. The aim of this paper is to estimate the long-term costs of CRC care by stage at diagnosis and phase of care in the Spanish National Health Service.

**Methods:**

Retrospective study on resource use and direct medical cost of a cohort of 699 patients diagnosed and treated for CRC in 2000–2006, with follow-up until 30 June 2011, at Hospital del Mar (Barcelona). The Kaplan-Meier sample average estimator was used to calculate observed 11-year costs, which were then extrapolated to 16 years. Bootstrap percentile confidence intervals were calculated for the mean long-term cost per patient by stage. Phase-specific, long-term costs for the entire CRC cohort were also estimated.

**Results:**

With regard to stage at diagnosis, the mean long-term cost per patient ranged from €20,708 (*in situ*) to €47,681 (stage III). The estimated costs increased at more advanced stages up to stage III and then substantially decreased in stage IV. In terms of treatment phase, the mean cost of the initial period represented 24.8 % of the total mean long-term cost, whereas the cost of continuing and advanced care phases represented 16.9 and 58.3 %, respectively.

**Conclusions:**

This study is the first to provide long-term cost estimates for CRC treatment, by stage at diagnosis and phase of care, based on data from clinical practice in Spain, and it will contribute useful information for future studies on cost-effectiveness and budget impact of different therapeutic innovations in Spain.

## Background

In Europe, colorectal cancer (CRC) represents the second most common type of cancer (13.0 % in the total number of incident cancers) [1]; in Spain, it ranks first in incidence (15.0 %) and second in both male and female mortality, after lung and breast cancer, respectively.

In recent years, new drugs such as bevacizumab, cetuximab and panitumumab have been developed for treatment of advanced CRC, promising potential improvements in patient outcomes [2, 3]. The introduction of these new drugs has increased the economic burden of CRC care, raising questions about the viability of its coverage in the public system [4, 5]. Thus, there is increasing interest in quantifying and evaluating the long-term costs by stage at diagnosis and phase of care. The assessment of long-term cost of CRC allows a better understanding of the disease burden and provides a useful source of information for cost-effectiveness studies on different preventive or alternative treatment initiatives.

Most studies that provide estimates of the long-term cost of CRC were conducted in the United States [6–9], but to our knowledge, there are no such studies in Spain. Because of differential organisation of health systems and different practice patterns and settings, the transferability of country specific results is not always possible, so further studies specific to Spain are needed. Because recently introduced drugs have significantly increased the cost of the treatment, some studies in Spain have focused on chemotherapy treatments or on a particular phase of the disease [10, 11]. Cots et al. [12] examined the costs of CRC treatment by stage at diagnosis and phase of care but did not take into account patient loss during the follow-up period, nor did they have complete survival information for the cases included in the analysis. Despite this limitation, their analysis provides a basis for long-term cost estimation. The aim of this paper is to estimate the long-term costs of CRC care by stage at diagnosis and phase of care in the Spanish National Health Service context.

## Methods

### Study design

Retrospective cohort study of 699 patients diagnosed and treated for CRC from 2000–2006 at Hospital del Mar (Barcelona), with follow-up until 30 June 2011. A prospective analysis of survival and long-term costs was also performed. The Hospital del Mar belongs to the Public Use Hospital Network of Catalonia.

### Data sources

A database was built linking different data sources related to CRC care: the Hospital Cancer Registry, the clinical-administrative information system and the cost accounting system. The Hospital Cancer Registry identified the CRC cases, their TNM stage at the time of diagnosis (TNM classification of the American Joint Committee on Cancer) [13] as well as the date and cause of death. The TNM system is based on the size and/or extent of the primary tumour (T), the amount of spread to nearby lymph nodes (N), and the presence of metastasis (M). In turn, information on the date and cause of death is obtained annually through a record linkage procedure between the Hospital Cancer Registry and the Catalonian Mortality Registry. The clinical-administrative information system provided information on health care episodes (inpatient discharges, surgeries, outpatient visits, chemotherapy and radiotherapy sessions) and health care services related to them (length of stay in conventional inpatient care units, length of stay in intensive care unit, operating room, doses of chemotherapy drugs, antiemetic and other related drugs, outpatient dispensing drugs, number and complexity of radiotherapy sessions, laboratory and radiology tests). Unit costs were obtained from the cost accounting system, which is an Activity Based Costing analytical accounting system implemented over 20 years ago.

This study was performed in accordance with the ethical standards of the Declaration of Helsinki and complied with the legal regulations on data confidentiality (*Ley Orgánica 15/1999, de 13 de diciembre, de Protección de Datos de Carácter Personal*). It was also approved by the Ethics Committee of Hospital del Mar.

### Attributable cancer-related cost

It is difficult to discern from administrative databases which episodes of care (inpatient admission, outpatient or emergency visit) are clearly related to cancer care.

All episodes of care from the Medical Oncology, Radiotherapy, Gastroenterology, General and Digestive Surgery or Palliative Care Units were considered to be related to CRC treatment (and including comorbidities). All episodes of care related to the Internal Medicine Unit were included if this unit had diagnosed the disease.

### Cost histories assessment

To assess the cost of the episodes of care in the period considered, unit costs from 2005 were obtained from the cost accounting system. Cost values in this year were considered to be representative of the study period. Assigning the same year-specific cost unit to each healthcare service, any differences in cost over time would reflect changes in resource utilisation and not in price deviations arising from pharmaceutical companies’ policies or different production structures over the study period.

Taking into account the date of each care episode and service, monthly costs were assessed (cost histories). The year of diagnosis was considered the baseline year (Year 0) with costs from later calendar years being discounted at 3 % [14].

### Phase-specific costs

The time between diagnosis and death was divided into three phases: the *initial care phase* covered the time of diagnosis and the first course of therapy (surgery and/or adjuvant treatments). Its completion marked the transition from the initial care phase to the *continuing care phase*, which included the time of monitoring care following initial treatment. Finally, the *advanced care phase* began when local recurrence or metastasis appeared or when palliative treatments were administered. The transition to the advanced phase was determined by the confirmation of metastasis or recurrence by a hospital discharge report, the administration of advanced disease, palliative chemotherapy, radiotherapy treatment or admission into the Palliative Care Unit. An algorithm was developed in order to determine the duration of the phases and specific phase of care costs for each patient.

### Estimation of long-term costs

Long-term or lifetime costs are defined as the cumulative cost from the date of diagnosis to the date of death, but in long-term cost estimations, some patients are not actually followed until their death [15, 16], so these cost histories are censored. However, this introduces a problem: total cost is underestimated when based on the full sample of censored and non-censored cases, since patients who withdraw from the study, or who remain alive at the end of the follow-up period, will continue to incur costs after the study is over. On the other hand, if long-term cost is only estimated for patients with uncensored costs, the estimator is biased towards patients with shorter survival times because longer survival times are more likely to be censored. Therefore, censored data can lead to biased estimates if the appropriate analysis techniques are not used.

To account for censored data, some researchers have applied the standard survival analyses techniques [17]. The Kaplan-Meier method applied to cost-to-event analysis (where costs are treated as potentially right-censored survival times) leads to bias because the requirement of independent censoring times and death times may not be fulfilled [18, 19]. For this reason, a number of alternative techniques have been proposed in the literature for estimating mean total costs in the presence of censoring [16, 18, 20, 21].

In this analysis, patient records were collected from the period 2000–2006 and were followed up through 2011, for a maximum of 11 years of evaluation, a time horizon unlikely to be adequate for evaluating long-term costs among patients diagnosed with non-advanced cancer. Thus, modeling techniques for the analysis of censored cost data were used to estimate long-term health care costs attributable to CRC [20, 22]. This estimation was carried out in two steps. First, long-term costs of CRC care were estimated for the 2000–2011 study period directly from observable data. We then extrapolated the estimated costs, adding 5 years from observed cost data (i.e. years 11 to 16), taking into account the care phases.

Costs for years 1 to 11 were estimated using the nonparametric Kaplan-Meier Sample Average (KMSA) estimator, which takes into account the censorship of survival and cost data. This method assumes that detailed patient cost histories are available and makes use of this information when estimating the mean total cost. The KMSA estimator computes costs by adding up expected costs incurred during each time interval, calculated as the product of the probability of surviving to that time interval (Kaplan-Meier estimates of survival), and the average sample cost among patients who survive to the beginning of the interval.

Let *C* be the average total cost for treating patients with CRC over the study period. We divide the entire time period into *K* survival times [*t*_*j*_, *t*_*j* + 1_) of one-month duration each. Since the cost histories are recorded, *C* can be decomposed as C_1_…C_K_.

The Kaplan-Meier estimator of the probability of surviving to time (month) *j* is given by

$$ {\overset{\frown }{S}}_j={\displaystyle \prod_{j:{t}_j\le t}\frac{n_j-{d}_j}{n_j}} $$

Where *n* is the number of subjects, with survival time at *t*_*j*_, with *d*_*j*_ being the number of deaths at *t*_*j*_, and *n*_*j*_ being the number of subjects at risk at *t*_*j*_.

The estimate of the average total cost is given by

$$ {\overset{\frown }{C}}_1={\displaystyle \sum_j{C}_j{S}_j} $$

Where *C*_*j*_ is the observed mean cost in month *j* among survivors to month *j* and *S*_*j*_ is the disease-specific survival probability at month *j*. This estimator takes into account the information of each patient at every observed month.

Costs for years 11 through 16 were extrapolated, based on the assumption that cohort-specific, average annual costs were constant for years beyond the available data until the year before the final year of life (continuing costs). These continuing phase costs were estimated as the average annual continuing phase cost from the subset of patients in each stage who were not considered as outliers (lying beyond the two standard deviations of the geometric mean). In addition, we assumed that medical care costs in the advanced care phase were the same for patients who lived beyond the 11-year study period, regardless of time from diagnosis; advanced care or terminal costs were thus estimated as the annualised average advanced-care phase cost among patients in the relevant stage who died of CRC.

We estimated the extrapolated average total cost given by

$$ {\overset{\frown }{C}}_2={\displaystyle \sum_{y=11}^{16}\Big\{{d}_y{C}_{dy}+\left(1-{d}_y\right){C}_{sy}}\Big\}{S}_y $$

Where *d*_*y*_ is the hazard of dying at year *y*, *C*_*dy*_ is the estimation of the annual terminal cost in year *y*, *C*_*sy*_ is the estimation of the annual cost in the continuing phase in year *y*, and *S*_*y*_ is the disease-specific survival probability at year *y*. The annual hazard of dying and the survival probability in years 11 to 16 were estimated from Weibull models of survival. Finally, long-term cost estimates were obtained by adding ${\overset{\frown }{C}}_1$ and ${\overset{\frown }{C}}_2$.

Long-term costs were reported by TNM stage at the time of diagnosis. Bootstrap percentile confidence intervals were calculated for the mean long-term cost per patient by stage at diagnosis from re-sampling the data 10,000 times. All analyses were done through R 3.1.0 software.

### Phase-specific cost estimates for the entire CRC cohort

Long-term cost estimation required calculating continuing care and advanced phase costs for CRC patients who survived at least 11 years. For completeness, phase-specific long-term costs for the entire CRC cohort were also estimated. For patients living fewer than 11 years from diagnosis, specific phase of care costs were estimated by means of the clinical algorithm developed in order to determine the duration of the phases for each patient.

## Results

### Patient characteristics and survival

A total of 699 cases of CRC were analysed, of which 512 (73.2 %) were diagnosed with colon cancer and 187 (26.8 %) were diagnosed with rectal cancer. Table 1 presents age, gender, staging distribution of patients at diagnosis, and treatments received. Table 2 shows the distribution of deaths, mean survival time and mean follow-up time by TNM stage at diagnosis. The mean survival time decreased from *in situ* cases to stage IV diagnoses, ranging from 137.5 to 21.2 months.

**Table 1:** Patient characteristics

	Colon	Rectum	Total
N	512	187	699
Age (years)			
Mean	70.4	70.5	70.5
Median	73.0	72.0	73.0
Sex (%)			
Male	53.9	59.4	55.4
Female	46.1	40.6	44.6
Year of diagnosis (%)			
2000	13.3	12.3	13.0
2001	14.3	15.5	14.6
2002	15.0	14.4	14.9
2003	17.0	13.4	16.0
2004	17.0	13.9	16.2
2005	11.5	12.8	11.9
2006	11.9	17.6	13.4
TNM Stage (%)			
*In situ*	6.6	3.2	5.7
I	11.7	14.4	12.5
II	25.8	25.1	25.6
III	23.2	29.4	24.9
IV	21.3	18.7	20.6
Unknown	11.3	9.1	10.7
Surgery (%)			
Patients	68.9	75.4	70.7
Surgical procedure			
Right hemicolectomy	27.7	1.1	18.6
Left hemicolectomy	5.5	0.7	3.8
Sigmoidectomy	18.9	1.1	12.8
Other colon resections	9.2	2.2	6.8
Anterior resection of rectum	3.9	27.7	12.1
Abdominoperineal resection	0.0	14.2	4.9
Other rectal resections	0.8	10.9	4.2
Liver metastases resection	3.5	3.4	3.5
Other metastases resection	1.8	2.2	1.9
Other procedures	28.8	36.3	31.4
Chemotherapy (%)			
Patients	30.1	47.1	34.6
Intention			
Neoadjuvant	1.4	27.7	11.0
Adjuvant	29.0	26.2	28.0
Advanced disease - Palliative	63.7	44.1	56.6
Unknown	5.9	2.0	4.5
Treatment scheme			
Fluorouracil +/− Levamisole	20.0	43.9	28.5
Capecitabine +/− Irinotecan/Oxaliplatin	23.8	16.6	21.3
Irinotecan - Fluorouracil +/− Leucovorin	14.7	17.1	15.6
Oxaliplatin - Fluorouracil +/− Leucovorin	12.1	7.5	10.4
Cetuximab - Irinotecan	10.3	3.2	7.8
Others	19.1	11.8	16.5
Radiotherapy (%)			
Patients	7.2	48.7	18.3
Intention			
Radical	39.5	74.5	64.4
Palliative	60.5	25.5	35.6

**Table 2:** Survival time by TNM stage at diagnosis

TNM Stage	N	Deaths	Censored cases	Survival time (months)	Follow-up time (months)
		N (%)	N (%)	Mean [SD]	Mean [SD]
*In situ*	40	-	40 (100.0)	137.5 [−]	56.6 [34.3]
I	87	8 (9.2)	79 (90.8)	126.1 [35.9]	73.9 [35.0]
II	179	53 (29.6)	126 (70.4)	98.7 [59.4]	56.4 [40.3]
III	174	80 (46.0)	94 (54.0)	80.1 [61.2]	50.3 [37.0]
IV	144	118 (81.9)	26 (18.1)	21.2 [39.2]	14.4 [21.3]
Unknown	75	33 (44.0)	42 (56.0)	68.7 [73.7]	28.3 [38.4]
Total	699	292 (41.8)	407 (58.2)	82.3 [66.6]	45.4 [39.9]

*SD* standard deviation

### Long-term costs estimates

**Fig. 1:**
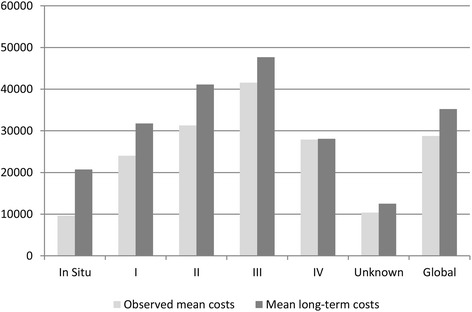
Observed mean cost and and mean long-term cost by TNM stage at diagnosis (€, 2005)

Table 3 shows the observed mean cost, KMSA estimation, cost extrapolation and mean long-term cost by TNM stage at diagnosis. Observed mean cost per patient ranged from €9634 among in situ patients to €41,550 in stage III. The KMSA estimator showed an estimated total cost that ranged from €17,692 among *in situ* cases to €44,934 among patients diagnosed at stage III. Taking into account the extrapolation up to five years beyond the observed costs, the estimated mean long-term costs ranged from €20,708 among *in situ* cases to €47,681 for stage III. In all cases, the estimated costs increased in more advanced stages up to stage III and then decreased substantially in stage IV. Figure 1 shows the observed mean costs over the first 11 years and the total long-term costs until year 16 after diagnosis by stage. The difference between the observed costs and estimated long-term costs decreased in cases diagnosed at advanced stages.

**Table 3:** Observed mean cost, KMSA estimation, cost extrapolation and mean long-term cost by TNM stage at diagnosis (€, 2005)

TNM Stage	N	Censored cases	Observed mean cost	KMSA estimation cost	Cost extrapolation	Total long-term costs	
		N (%)				Mean	95 % CI
*In situ*	40	40 (100.0)	9634	17,692	3016	20,708	(16,902; 25,500)
I	87	79 (90.8)	23,999	27,272	4485	31,757	(27,172; 36,731)
II	179	126 (70.4)	31,262	37,247	3869	41,116	(38,313; 43,979)
III	174	94 (54.0)	41,550	44,934	2747	47,681	(43,439; 52,551)
IV	144	26 (18.1)	27,873	28,050	10	28,061	(26,533; 29,694)
Unknown	75	42 (56.0)	10,363	11,976	521	12,497	(10,411; 14,842)
Global	699	407 (58.2)	28,741	32,772	2435	35,207	(33,521; 36,989)

*KMSA* Kaplan-Maier sample average, *CI* confidence interval

Table 4 shows the long-term costs of CRC treatment by TNM stage at diagnosis and phase of care. The mean cost of the initial care phase represented 24.8 % of the total mean long-term cost, whereas the cost of continuing and advanced phases represented 16.9 and 58.3 %, respectively. Higher costs were found during the initial care and advanced care phase in all stages, with the exception of cases diagnosed *in situ* and at stage I, where the mean cost of the continuing care phase represented 56.0 and 50.5 % of the total mean long-term cost, respectively.

**Table 4:** Long-term cost of CRC treatment by TNM stage at diagnosis and phase of care (€, 2005)

TNM Stage	N	Initial phase		Continuing phase		Advanced-care phase		Total long-term cost	
		Mean	(%)	Mean	%	Mean	%	Mean	%
*In situ*	40	3852	(18.6)	11,593	(56.0)	5263	(25.4)	20,708	(100.0)
I	87	11,104	(35.0)	16,038	(50.5)	4615	(14.5)	31,757	(100.0)
II	179	13,251	(32.2)	11,100	(27.0)	16,764	(40.8)	41,116	(100.0)
III	174	14,182	(29.7)	6524	(13.7)	26,975	(56.6)	47,681	(100.0)
IV	144	1429	(5.1)	-	ᅟ	26,632	(94.9)	28,061	(100.0)
Unknown	75	1119	(9.0)	514	(4.1)	10,863	(86.9)	12,497	(100.0)
Global	699	8740	(24.8)	5945	(16.9)	20,522	(58.3)	35,207	(100.0)

The initial and continuing care phase costs decreased in relative weight as disease stage progressed. The initial phase costs ranged from 35.0 % of the total at stage I to 5.1 % at stage IV, and the continuing care phase from 50.5 % at stage I to 0 % at stage IV. Moreover, the relative cost of the advanced care phase increased for diagnoses in more advanced stages, from 14.5 % at stage I to 94.9 % at stage IV.

## Discussion

Long-term costs of CRC care were estimated in a cohort of 699 patients diagnosed and treated for CRC in Spain in 2000–2006, with follow-up until 30 June 2011. Total mean long-term cost ranged from €20,708 among *in situ* cases, to €47,681 among patients diagnosed at stage III. The estimated costs increased in more advanced stages up to stage III and then decreased substantially in stage IV. Advanced disease stages were associated with a decrease in the relative weight of the cost of the initial and continuing care phases, but an increase in advanced disease cost. As expected, the difference between the mean observed costs and estimated mean long-term costs decreased in advanced stages, reflecting the shorter survival time of patients diagnosed with advanced tumours.

In the last fifteen years, various studies have estimated the long-term cost of CRC treatment in the international context, with different objectives, methodologies and health contexts [18, 22–31]. Some studies have looked at the estimated cost per patient showing a positive trend as disease stage progressed [23, 24, 26, 28, 31]. This association remained positive up to stage III, with a decrease in stage IV, mainly due to the lower weight of surgery costs at this stage. In addition, the high costs for patients diagnosed at earlier stages reflect increased medical costs during the initial care phase, together with longer survival times compared to patients diagnosed at more advanced stages. However, in the studies carried out by Etzioni et al. [22] and Lang et al. [30], mean lifetime cancer-related costs decreased as disease stage progressed. In these studies, costs associated with CRC were estimated as the difference in the total costs of all medical care between matched cancer patients and control patients. They found that the excess lifetime costs for stage IV cases were very low, reflecting the fact that increased costs from the time of diagnosis were offset by their low survival and the costs for controls over their additional years of life. In any case, the results of these two studies are somewhat lower than ours or those from Brown et al. [23] because unrelated future medical costs are accounted for in the total lifetime projected cost.

In line with what was found in different studies [23–25], higher costs were found during the initial care phase (basically due to surgical procedures) and the advanced care phase due to hospitalisations, chemotherapy, and palliative care, with the exception of patients diagnosed in situ and at stage I, where the continuing care phase represented almost 50 % of total mean lifetime cost. Maroun et al. [24] found that initial treatment accounted for 49 % of the total cost, while 28 and 27 % of the total cost pertained to the terminal phase for colon and rectum cancers, respectively. Our results show that, in stages *in situ*, I and II, the mean cost of the initial phase was higher than in the advanced phase, while the cost of the initial phase was lower in stages III and IV. In the study of Lang et al. [30], the mean cost of the initial phase was greater than the advanced phase regardless of stage at diagnosis.

Most long-term cost studies of CRC have been conducted in the United States [6–9], but to our knowledge, there are no such studies in Spain. Despite the recognised need for specific studies in Spain, there is a lack of appropriate sources of information to perform these studies. There are not many hospitals with key information sources to assess hospital costs of cancer care: a hospital cancer registry, clinical-administrative information system and analytical accounting system. Cots et al. [12] examined the costs of CRC treatment for a cohort of patients diagnosed with CRC in Hospital del Mar in 2000 by stage at diagnosis and phase of care. A cost methodology based on clinical information was used, but it was a retrospective study that did not take into account patient withdrawals during the follow-up period, and it did not have complete survival information on the cases included in the analysis. Despite this limitation, this analysis provided a basis for a long-term cost estimation. Because recently introduced drugs have significantly increased treatment costs, some studies have focused on chemotherapy treatments or on a particular phase of the disease [10, 11].

The methodology used in this study combines a non-parametric evaluation of observed data over the initial years post-diagnosis with model-based estimates for those who survive beyond the period of data observation. This methodology differs from that used in the American studies, where the costs associated with CRC were estimated from the difference in the total costs of all medical care between matched cancer patients and control patients [18, 22, 23, 25, 27, 28, 30–32]. Therefore, the estimated attributable costs reflect the excess costs for CRC patients relative to a set of matched controls, rather than strictly the disease-related or attributable costs.

Another difference with these studies is the definition of the phases of care. In previous studies, the duration of care phases was fixed and equal for all patients (6/12 months for the initial care phase, and the last 12 months for the advanced care phase). The definition of phases was based on the observed U-shaped patterns of costs following diagnosis in a previous study (highest costs in the initial and terminal phases of care and lower costs in the continuing phase of care) [33]. These phases were chosen to coincide with clinically relevant events. In our study, the duration of the three phases for each patient was determined through an algorithm based on clinical information, which can reflect and predict disease progression more accurately. This approach could affect the phase of care cost estimation obtained (e.g. the cost of the advanced phase of care, with a longer duration, was greater in all stages compared to the estimation obtained through the last 12 months of life; data not shown).

This study is also strengthened by the sources of information used to measure costs. By contrast, most other studies assessed heath care costs by reviewing expenses or charges made to patients, rather than actual costs [32].

Still, our results should be interpreted in light of the model assumptions and their potential limitations. Some of these limitations are common to all studies that rely on clinical-administrative databases, such as potential coding errors and incomplete data. Moreover, current higher complexity chemotherapy treatments were not included. The study design required a long follow-up period, complicating the consideration of recent treatments in the analysis. In any case, this fact would increase the cost of the treatment of CRC.

Another limitation is that our results respond to the organisational characteristics and clinical practice of one centre, which limits the generalisability of the study findings. However, the Hospital del Mar is a public acute care reference hospital in the treatment of cancer, which follows the clinical guidelines of the Spanish National Health System. Moreover, only a few hospitals in Spain have the essential information sources to carry out this type of studies: a hospital cancer registry, clinical-administrative information system and analytical accounting system. Further studies should be conducted adapting our methods to other patient populations and settings.

## Conclusions

This study provides long-term cost estimates for CRC treatment by stage at diagnosis and phase of care. Estimated costs increased as disease stage progressed up to Stage III. The highest costs were found during the initial and advanced care phases, with the exception of *in situ* and stage I patients, where the mean cost of continuing phase represented almost 50 % of total mean long-term cost. As far as we know, ours is the first study that provides long-term costs for CRC treatment based on clinical practice in Spain. This cost analysis is a baseline study that will provide a useful source of information for future studies on cost-effectiveness and on the budget impact of different therapeutic innovations in Spain.

## Abbreviations

CI: Confidence interval
CRC: Colorectal cancer
KMSA: Kaplan-Meier sample average
SD: Standard deviation
